# Case Report: Lithium-induced bradycardia triggered by cecal volvulus: a case of obstruction-associated drug accumulation

**DOI:** 10.3389/fgstr.2026.1728396

**Published:** 2026-10-01

**Authors:** Akram Alnounou, Henry Zou, Madison Laird, Christopher Visak, Sage Bilsland, Sophie Numbers, Neil Hughes

**Affiliations:** Western Michigan University Homer Stryker MD School of Medicine, Kalamazoo, MI, United States

**Keywords:** bowel obstruction, bradycardia, encephalopathy, lithium, volvulus

## Abstract

**Background:**

Lithium toxicity can rarely manifest as cardiac arrhythmias, often initially presenting as bradycardia. Bowel obstruction is a rare cause of acute lithium toxicity. We present a case of lithium-induced symptomatic bradycardia induced by cecal volvulus.

**Case report:**

An 84-year-old female with a history of bipolar 1 disorder on chronic lithium therapy was admitted for symptomatic bradycardia and abdominal pain. She also presented with myoclonus, polydipsia, and encephalopathy. Laboratory evaluation revealed lithium toxicity in the setting of renal impairment, and abdominal imaging findings indicated large bowel obstruction secondary to cecal volvulus. Lithium was discontinued, and the patient was managed supportively. Despite declining lithium levels, the patient’s symptomatic bradycardia persisted until surgical correction of the volvulus, after which cardiac and neurological function markedly improved.

**Significance:**

This case highlights the potential role of gastrointestinal obstruction in exacerbating lithium-induced bradycardia by impairing its clearance and inducing a vagal response triggered by abdominal distension.

## Introduction

Lithium is widely used in the management of bipolar disorder but has a narrow therapeutic index and potentially life-threatening complications when serum levels rise above 1.5 mEq/L (1). Cardiac manifestations, although uncommon, include sinoatrial node dysfunction, atrioventricular block, and ventricular arrhythmias, with bradycardia often being the initial presentation (2). Gastrointestinal disturbances and bowel dysmotility can exacerbate lithium accumulation by promoting dehydration and reducing renal clearance (3). Gastrointestinal obstruction, including bezoars or malignancy, has been reported as a rare trigger of acute lithium toxicity (4, 5). We present a case of lithium-induced symptomatic bradycardia induced by large l bowel obstruction caused by cecal volvulus.

## Case presentation

An 84-year-old female with a history of chronic kidney disease stage 3 (CKD3), bipolar I disorder treated with lithium and hyperlipidemia was admitted for symptomatic bradycardia and intermittent, non-radiating, bandlike right lower abdominal pain associated with loose stools and decreased oral intake for three days. Approximately one month prior to admission, the patient developed progressively worsening tremors and myoclonic jerks, particularly evident during fine motor tasks such as writing. She also experienced intermittent dizziness, lightheadedness, and increasing fatigue. Her family reported increased water intake and episodes of slurred speech and altered mental status. Her home medications included lithium, which had been managed by her primary care physician with regular monitoring of renal function, with creatinine levels steadily increasing from 0.95 to 1.24 mg/dL.

On presentation, the patient was alert but showed signs of mild encephalopathy. Vital signs were notable for bradycardia (30–49 bpm). Abdominal exam revealed tenderness in the right lower quadrant without peritoneal signs. The neurological exam demonstrated resting tremor and occasional myoclonic jerks but no focal deficits. Mental status was slowed but oriented to self, place, and time.

Initial electrocardiogram (ECG) showed idioventricular rhythm and left bundle branch block. Laboratory evaluation revealed hypercreatininemia (1.43 mg/dL), non-anion gap metabolic acidosis, leukocytosis, and hypernatremia (149 mmol/L). Lithium level on admission was 1.6 mmol/L. Abdominal X-ray revealed severely distended bowel loops initially concerning for small bowel obstruction (**Figure 1**), but subsequent abdominopelvic computed tomography (CT) showed an 8.6 cm diameter transversely-oriented distended cecum (**Figure 2**). CT of the brain showed chronic microvascular changes but no acute pathology. Urinalysis was positive and urine culture grew *Streptococcus dysgalactiae*.

**Figure 1:**
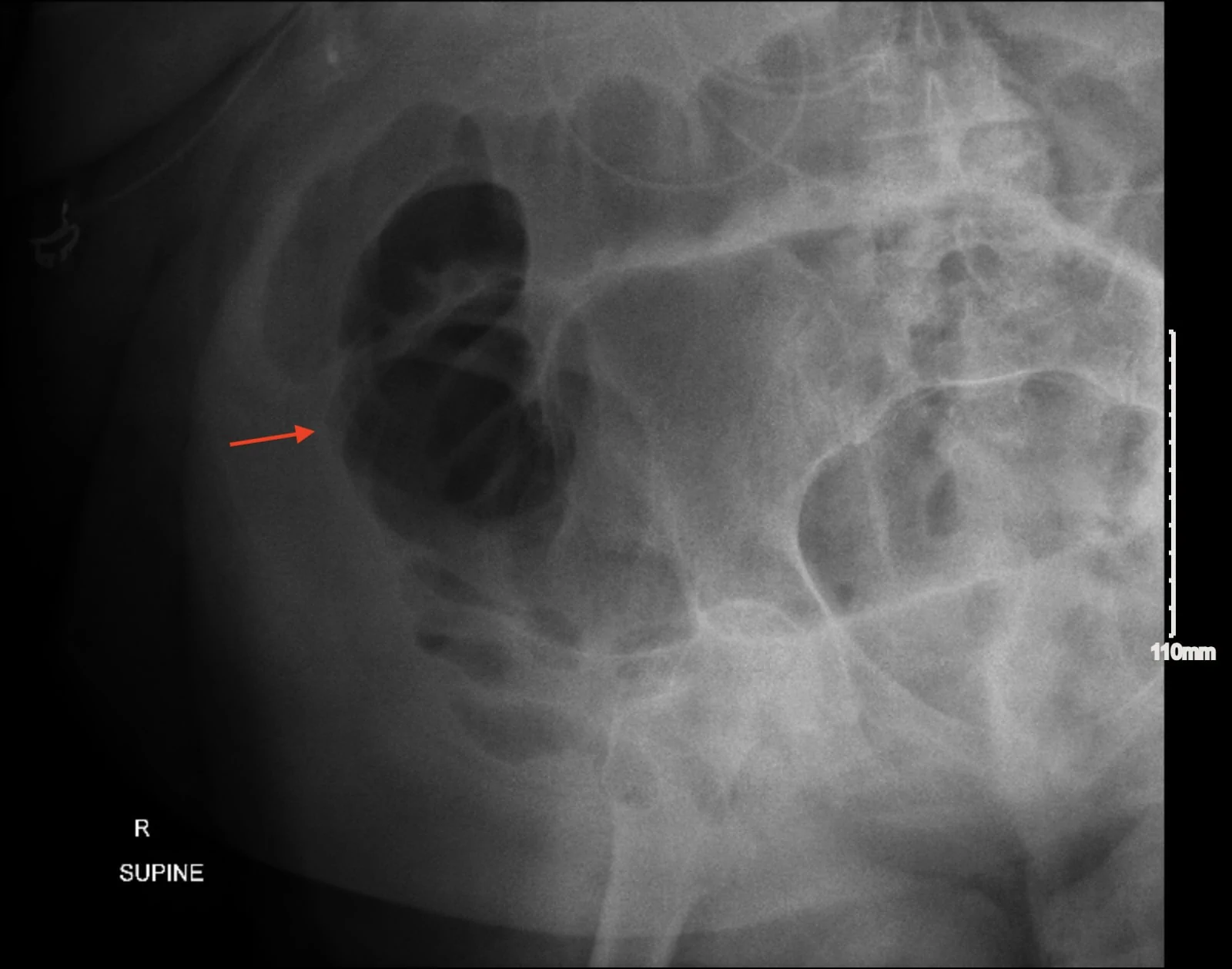
Severe bowel loop distension on abdominal X-ray (red arrow).

**Figure 2:**
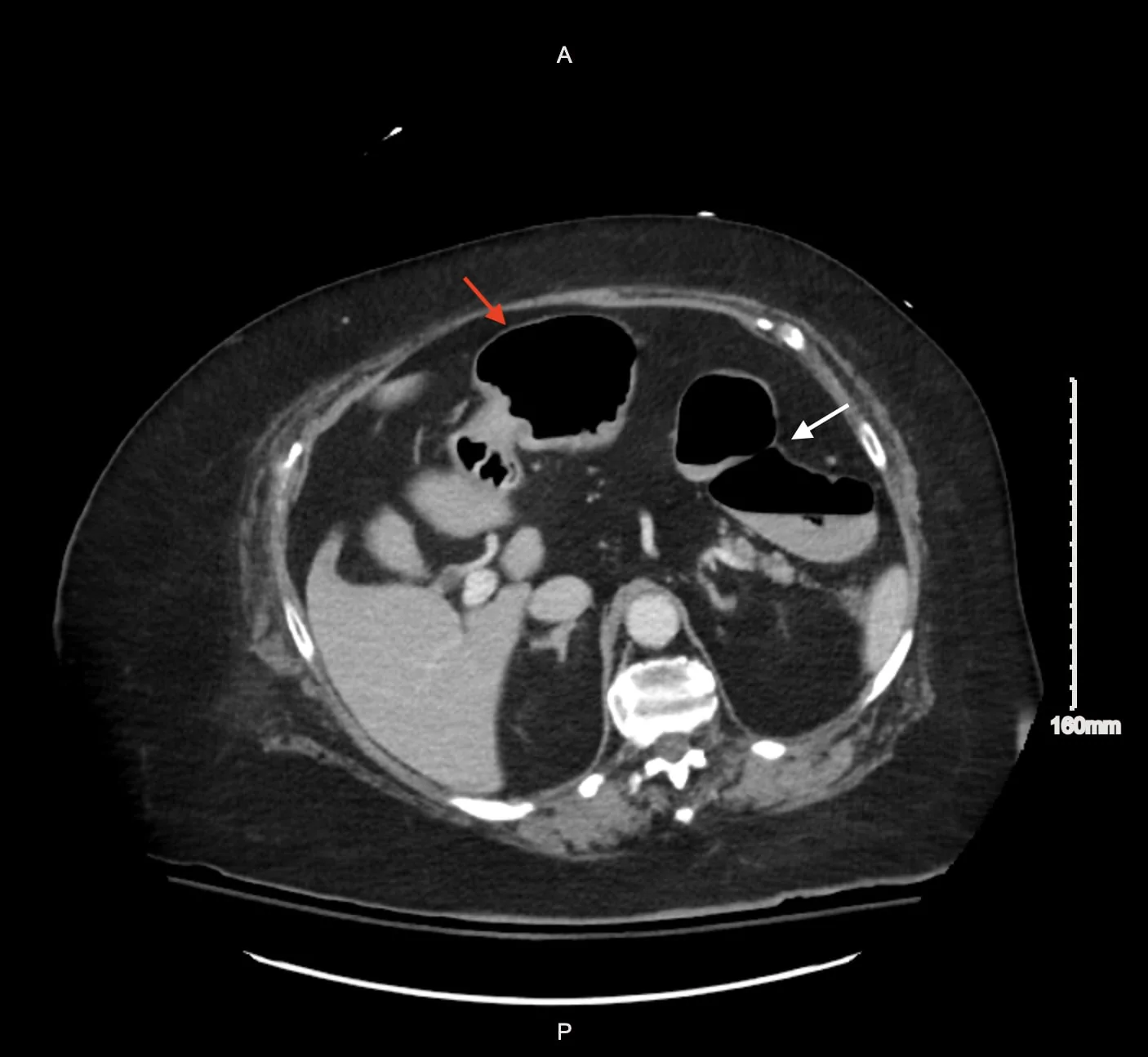
Transversely-oriented cecal distension on abdominopelvic CT (red arrow) with diffuse transverse and descending colon air distension (white arrow).

The patient’s progressive tremor, myoclonus, encephalopathy, chronic lithium use, worsening renal function, and elevated serum lithium concentration were considered most consistent with acute-on-chronic lithium toxicity. The differential diagnosis for her symptomatic bradycardia included lithium toxicity, sinus node dysfunction or atrioventricular conduction disease, metabolic derangements, infection-related bradycardia, and increased vagal tone secondary to bowel obstruction. Although the urinary tract infection and chronic kidney disease may have contributed to her altered mental status and impaired lithium clearance, they did not adequately explain the constellation of neurologic findings or profound bradycardia. Additionally, the persistence of abdominal pain despite supportive care prompted further evaluation, and CT findings established cecal volvulus as an additional diagnosis likely contributing to both impaired lithium elimination and increased vagal tone.

Based on the patient’s chronic lithium use, progressive neurologic symptoms, elevated serum lithium concentration, worsening renal function, and imaging findings, acute-on-chronic lithium toxicity complicated by cecal volvulus was considered the most likely diagnosis. The patient was admitted for supportive care of suspected acute-on-chronic lithium toxicity and symptomatic bradycardia. Lithium was discontinued and aggressive intravenous (IV) hydration initiated, with transition to 5% dextrose-in-water (D5W) for hypernatremia management. Nephrology was consulted and recommended close sodium and creatinine monitoring, as well as initiation of amiloride for suspected lithium-induced arginine vasopressin (AVP) resistance. Cardiology advised against pacemaker placement given the presumed reversible etiology of bradycardia. Due to radiographic evidence of cecal volvulus and worsening abdominal distension (**Figure 3**), General Surgery performed an exploratory laparotomy with right hemicolectomy and ileocolic anastomosis.

**Figure 3:**
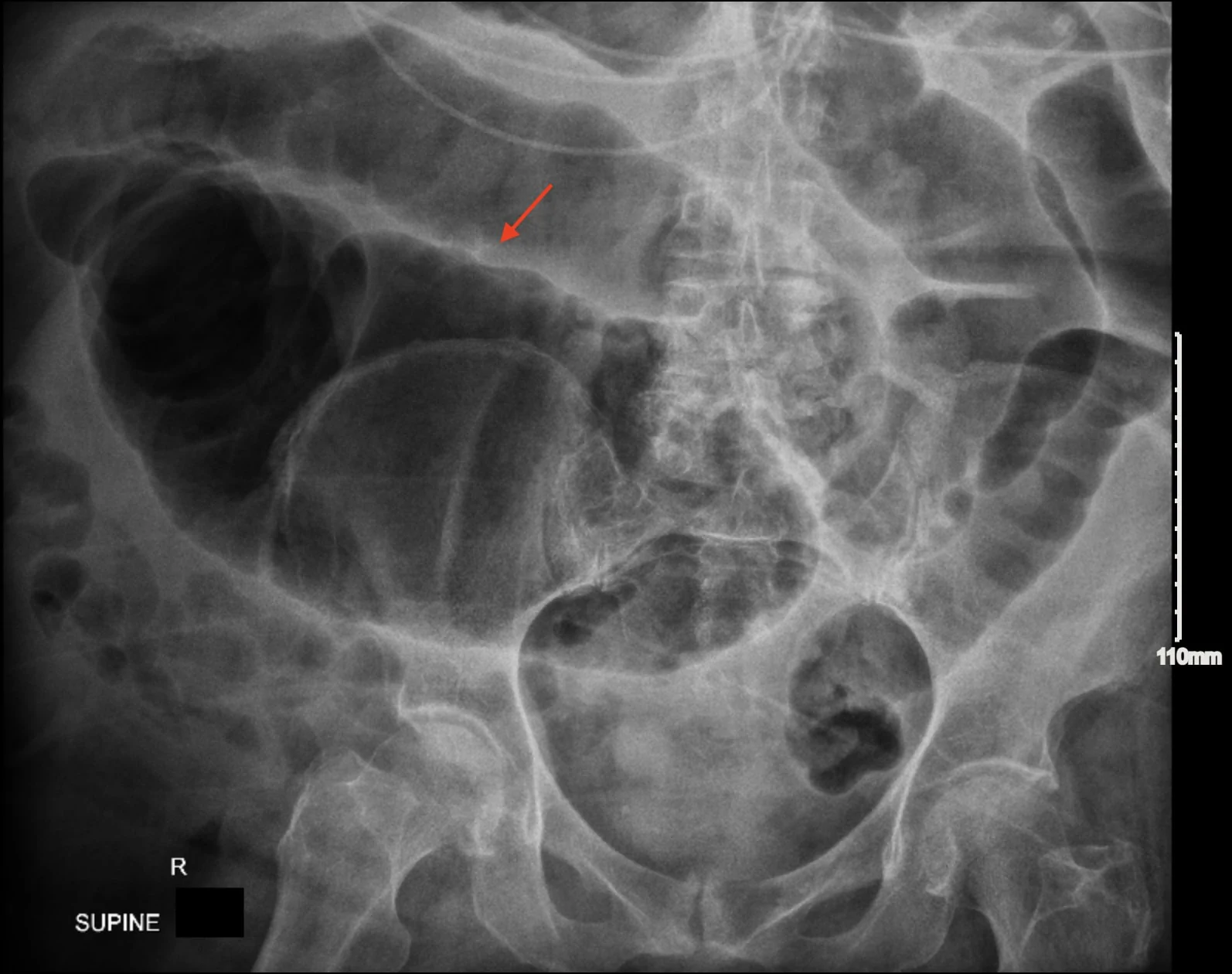
Interval worsening of cecal distension (red arrow) on abdominal X-ray.

The patient’s lithium level steadily declined, reaching 0.1 mmol/L by day 11. However, her encephalopathy and bradycardia persisted despite declining lithium levels. Following surgical correction of the bowel obstruction, the patient’s heart rate normalized and neurologic symptoms improved markedly. Postoperative ileus resolved with conservative management (IV hydration, antiemetics, and slowly advancing a liquid diet). She was discharged in stable condition with resolved encephalopathy and normal sinus rhythm (79 bpm). The clinical course, diagnostic findings, and management are summarized in (**Table 1**).

**Table 1:** Timeline of clinical course, diagnostic findings, and management.

Time point	Clinical findings	Key diagnostic data	Management	Clinical outcome/relevance
Approximately 1 month before admission	Progressive tremor, myoclonic jerks, dizziness, fatigue, slurred speech, altered mental status, and increased water intake while on chronic lithium therapy.	Creatinine rose from 0.95 to 1.24 mg/dL during outpatient monitoring.	Renal function monitored by primary care while lithium therapy continued.	Progressive neurologic symptoms and renal impairment preceded hospitalization.
3 days before admission	Intermittent, non-radiating, bandlike right lower abdominal pain with loose stools and decreased oral intake.	Decreased intake raised concern for dehydration and reduced renal lithium clearance.	Patient presented for evaluation.	Abdominal symptoms preceded diagnosis of bowel obstruction.
Admission/hospital day 0	Symptomatic bradycardia, mild encephalopathy, resting tremor, myoclonus, and right lower quadrant tenderness without peritoneal signs.	Heart rate 30–49 bpm; lithium 1.6 mmol/L; creatinine 1.43 mg/dL; sodium 149 mmol/L; leukocytosis; non-anion gap metabolic acidosis. ECG showed idioventricular rhythm and left bundle branch block. Abdominal X-ray showed severe bowel loop distension.	Lithium discontinued. Aggressive IV hydration initiated. Nephrology and cardiology consulted.	Acute-on-chronic lithium toxicity was suspected as the initial unifying diagnosis.
Early hospitalization	Persistent bradycardia, encephalopathy, abdominal pain, and worsening abdominal distension despite supportive care.	Abdominopelvic CT showed an 8.6 cm transversely oriented distended cecum consistent with cecal volvulus. Brain CT showed no acute pathology. Urine culture grew Streptococcus dysgalactiae.	D5W used for hypernatremia management. Amiloride started for suspected lithium-induced AVP resistance. Pacemaker placement deferred because bradycardia was considered potentially reversible.	Cecal volvulus was identified as an additional driver of impaired lithium clearance and possible vagally mediated bradycardia.
Preoperative course	Bradycardia and encephalopathy persisted while abdominal distension worsened.	Repeat abdominal X-ray showed interval worsening of cecal distension. Serum lithium level continued to decline.	General Surgery proceeded with operative management.	Persistence of symptoms despite falling lithium level supported a multifactorial mechanism rather than serum lithium level alone.
Operative intervention	Cecal volvulus with progressive obstructive findings.	Radiographic evidence supported ongoing obstructive pathology.	Exploratory laparotomy with right hemicolectomy and ileocolic anastomosis.	Mechanical obstruction was corrected.
Postoperative course	Heart rate normalized and neurologic symptoms improved markedly after surgery.	Lithium level continued to decline, reaching 0.1 mmol/L by hospital day 11.	Postoperative ileus managed conservatively with IV hydration, antiemetics, and gradual diet advancement.	Improvement after surgical correction supported the role of obstruction and vagal physiology in persistent bradycardia.
Discharge	Encephalopathy resolved and cardiac rhythm stabilized.	Normal sinus rhythm with heart rate 79 bpm.	Discharged in stable condition with lithium discontinued.	Cardiac and neurologic abnormalities resolved.

AVP, arginine vasopressin; bpm, beats per minute; CT, computed tomography; D5W, 5% dextrose in water; ECG, electrocardiogram; IV, intravenous.

## Discussion

Lithium is a widely used treatment for bipolar disorder, but has a narrow therapeutic window (6). Lithium accumulation can lead to multiorgan dysfunction involving neurological, renal, gastrointestinal, endocrine, and rarely cardiovascular manifestations (6). An important diagnostic challenge in this case was that the patient’s serum lithium concentration (1.6 mmol/L), while within the toxic range, appeared disproportionate to the severity of her neurologic and cardiovascular manifestations. In patients receiving chronic lithium therapy, serum lithium concentrations may not accurately reflect the severity of toxicity, and clinically significant toxicity can occur despite serum concentrations that appear only modestly elevated relative to the patient’s clinical presentation (6). Therefore, the diagnosis of lithium toxicity, particularly in patients receiving chronic therapy, should be guided by the overall clinical presentation rather than serum lithium concentration alone.

This case also highlights the importance of sequential diagnostic reassessment. Although lithium toxicity was initially thought to explain the patient’s neurologic manifestations and bradycardia, the severity of her bradycardia appeared disproportionate to her modestly elevated serum lithium concentration, and its persistence despite declining lithium levels prompted further evaluation. Thus, the patient’s cecal volvulus may have contributed to her worsening cardiovascular abnormalities, supporting a multifactorial mechanism involving both impaired lithium clearance and vagally mediated bradycardia (8).

In our patient, we hypothesize that lithium-associated gastrointestinal dysmotility may have also contributed to conditions favoring cecal volvulus, although a predisposing anatomic abnormality likely also played a role (7). It is possible that the volvulus impaired lithium clearance, leading to further accumulation and worsening toxicity. Our patient’s presentation is similar to one of an 83-year-old woman who was diagnosed with lithium toxicity and hypermagnesemia induced by a colorectal cancer-associated obstructive ileus, which triggered severe bradyarrhythmia and hypotension (5). She recovered after fluid resuscitation, hemodialysis, and endoscopic stenting (5).

Our patient’s history of CKD3 and newly diagnosed AVP resistance may have also further contributed to her lithium toxicity (6). Gastrointestinal distension may also have independently contributed to the patient’s bradycardia by increasing vagal tone, which may explain why symptoms persisted despite normalization of serum lithium levels until surgical correction of her bowel obstruction was performed (8).

This case is inherently limited as a single case report and therefore cannot establish causality or determine the relative contributions of the hypothesized factors, including lithium toxicity, cecal volvulus, and gastrointestinal dysmotility to the patient’s severe bradycardia. However, this case highlights the complex, cyclical relationship between these factors and demonstrates how lithium accumulation in the setting of CKD and AVP resistance may contribute to gastrointestinal dysmotility and cecal volvulus, which can further impair lithium clearance and exacerbate toxicity, ultimately manifesting as symptomatic bradycardia. Clinicians should maintain a high level of suspicion for structural gastrointestinal issues in patients with lithium toxicity who present with unexplained or refractory bradycardia, as emergent surgical intervention may be indicated.

## Data Availability

The original contributions presented in the study are included in the article/supplementary material. Further inquiries can be directed to the corresponding author/s.
